# Enhanced Degradation of Naproxen by Immobilization of *Bacillus thuringiensis* B1(2015b) on Loofah Sponge

**DOI:** 10.3390/molecules25040872

**Published:** 2020-02-17

**Authors:** Anna Dzionek, Danuta Wojcieszyńska, Małgorzata Adamczyk-Habrajska, Urszula Guzik

**Affiliations:** 1 Institute of Biology, Biotechnology and Environmental Protection, Faculty of Natural Science, University of Silesia in Katowice, Jagiellońska 28, 40-032 Katowice, Poland; adzionek@us.edu.pl (A.D.); danuta.wojcieszynska@us.edu.pl (D.W.); 2 Institute of Materials Engineering, Faculty of Science and Technology, University of Silesia in Katowice, 75 Pułku Piechoty 1a, 41–500 Chorzów, Poland; malgorzata.adamczyk-habrajska@us.edu.pl

**Keywords:** bacteria, immobilization, loofah sponge, naproxen, trickling filter, wastewater treatment

## Abstract

The naproxen-degrading bacterium *Bacillus thuringiensis* B1(2015b) was immobilised onto loofah sponge and introduced into lab-scale trickling filters. The trickling filters constructed for this study additionally contained stabilised microflora from a functioning wastewater treatment plant to assess the behavior of introduced immobilized biocatalyst in a fully functioning bioremediation system. The immobilised cells degraded naproxen (1 mg/L) faster in the presence of autochthonous microflora than in a monoculture trickling filter. There was also abundant colonization of the loofah sponges by the microorganisms from the system. Analysis of the influence of an acute, short-term naproxen exposure on the indigenous community revealed a significant drop in its diversity and qualitative composition. Bioaugmentation was also not neutral to the microflora. Introducing a new microorganism and increasing the removal of the pollutant caused changes in the microbial community structure and species composition. The incorporation of the immobilised B1(2015b) was successful and the introduced strain colonized the basic carrier in the trickling filter after the complete biodegradation of the naproxen. As a result, the bioremediation system could potentially be used to biodegrade naproxen in the future.

## 1. Introduction

Pharmaceuticals and personal care products are nowadays being detected in surface water and groundwater more and more frequently. Although their concentration typically does not exceed 1 µg × L^−1^, chronic exposure on non-target organisms can have significant consequences. One of the drugs that can affect entire ecosystems is naproxen (2-(6-methoxy-2-naphthyl)propionic acid), which is a polycyclic non-steroidal anti-inflammatory drug (NSAID). This drug is not metabolized by humans and wastewater treatment plants do not have strains that are capable of degrading naproxen with high efficiency. Additionally, it is one of the drugs that is easily photolyzed. It has been proven that the naproxen phototransformation products are often more toxic than the drug itself [1]. It should also be noted that in the last phase of water purification in wastewater treatment plants sterilization using UV light is performed. Hence, not only is naproxen released into the environment, but also the products of its phototransformation.

Among the various bioremediation systems that are used in wastewater treatment plants, particular attention has been paid to those that are based on immobilised microorganisms. Naturally occurring biofilm and immobilization ensure the retention and accumulation of the biomass, which decreases the filtration costs. One of the systems that is based on this attached growth is a trickling filter. These systems are characterized by a simpler design and a smaller environmental footprint due to their lower installation and operational costs compared to other wastewater treatment systems [2]. As was mentioned above, most wastewater treatment plants are not adapted to remove NSAIDs. One possible solution might be bioaugmenting the existing bioremediation systems.

Introducing strains that are capable of utilising specific pollutants in bioremediation systems has many advantages as well as a few disadvantages. The biggest benefit is associated with removing the pollutants, which despite the use of low concentrations may have a toxic effect on autochthonous microflora. If bioaugmentation is successful and the introduced strains are not removed from the system, the biodegradation of the contaminants will remain constant over a long period of time. However, in order to make this possible, after the introduction, the strains must show a degradative activity, colonize the system and be able to propagate in it, which does not always occur. The success of bioaugmentation is determined by many factors. Not only can the selection of the strains with the appropriate features be crucial, but also the way that they are introduced into a complex community. In addition to preparing a sufficiently high biomass concentration, it is also important to conduct an acclimatization pre-run. Such a process should be performed in a separate system that is run under the same conditions but only with the strain that was selected for the bioaugmentation so that it can adapt to the conditions that prevail in the system [3]. To increase the chance of the survival of the introduced strains, they can also be introduced in an immobilised form. This method is fast, inexpensive and simple and does not require any acclimatization or specialized equipment. The most commonly used method for immobilization for bioaugmentation is adsorption on the surface because it results with a shaped biofilm that is introduced into the system. Additionally, immobilization provides a barrier that protects the introduced cells from other microorganisms and the substances that they excrete as well as from toxic shock or environmental fluctuations [4]. However, bioaugmentation can also have negative consequences. One of these is the significant changes in the composition of the autochthonous microflora that result from the competition and inhibition of the new strains. One consequence may be a significant drop in the effectiveness of the system [5]. For that reason, an experiment that examines the interactions between the immobilised strains that are introduced into wastewater treatment systems and the microbial communities that are present in these systems as well as the influence of these communities on the degradation capacity should be performed.

In our work, we present the process of developing an immobilised biocatalyst which was constructed to bioaugmentation of the bioremediation system and to investigate its naproxen degradation capabilities in such a system. A Gram-positive *Bacillus thuringiensis* B1(2015b), which is able to degrade naproxen under cometabolic conditions, was selected to be the introduced strain [6]. The cells were immobilised on the loofah sponge through adsorption on the surface and were introduced into a trickling filter that contained stable microbial communities from the wastewater treatment plant. To determine the influence of the autochthonous microflora that was present in the bioremediation system on the efficiency of naproxen biodegradation by the immobilised B1(2015b), the removal of the drug in the bioaugmented trickling filter and in a system without autochthonous microflora was monitored. We present a visualization of the biofilm that was created on the loofah sponges and its colonization after its introduction into the trickling filter. Additionally, the impact of an acute, short-term naproxen exposure and bioaugmentation on the qualitative composition of the autochthonous microflora was also evaluated. This is the first report about wastewater treatment system bioaugmentation with immobilised cells that are capable of degrading naproxen that includes its impact on the autochthonous microflora.

## 2. Results

### 2.1. Immobilization of Bacillus thuringiensis B1(2015b) on Loofah Sponge

The immobilization process in this study was the most efficient in a HCT medium (pH 8) in the presence of glucose with shaking (110 rpm) at 20 °C for 48 h. A HCT medium is very rich in both carbon sources and metal ions and therefore it was used to form biofilm by the *Bacillus thuringiensis* genus [7]. Analysis of the initial amount of the cells shows that the smallest number of cells (OD_600_ equal to 0.2) resulted in the highest mass of the biofilm and the enzymatic activity. It was observed that the age of a culture that is used for immobilization has a significant influence on the metabolic activity of a biofilm. The lowest enzyme activity was demonstrated by a 24 h old biofilm. The highest activity was observed in 48 h and 72 h old cultures. That is why a 48 h old culture of B1(2015b) was selected for the immobilization process in this study. Interesting results we obtained during the analysis of the medium pH and addition of the different metal salts. A significant increase in the enzymatic activity of the biofilm was noted when incubation was conducted in pH 8.0 and with supplementation with manganese (Figure S1, Supplementary Materials).

After the optimization of each immobilization parameter immobilized biocatalyst contained 28 ± 3.5 mg of dry biofilm mass per loofah cube, which hydrolyzed fluorescein diacetate (FDA) to 19.07 ± 1.06 μg/mL of fluorescein in 1 h (Table 1). FDA abiotic hydrolysis and fluorescein adsorption by the loofah cubes that were not immobilised was not statistically significant.

### 2.2. Naproxen Biodegradation in the Trickling Filters

Trickling filters TF-I and TF-C were designed to recreate the conditions that prevail in wastewater treatment plants. The only distinguishing parameter was maintaining a constant, room temperature. The flow rate was adjusted to 0.0066 m^3^/h in order to prevent the wastewater from spraying through packing material too strongly or quickly. Simultaneously, this enables the time of contact between wastewater and microorganisms to be established, which is expressed as the hydraulic retention time (HRT), which was set for six hours.

**Figure 1:**
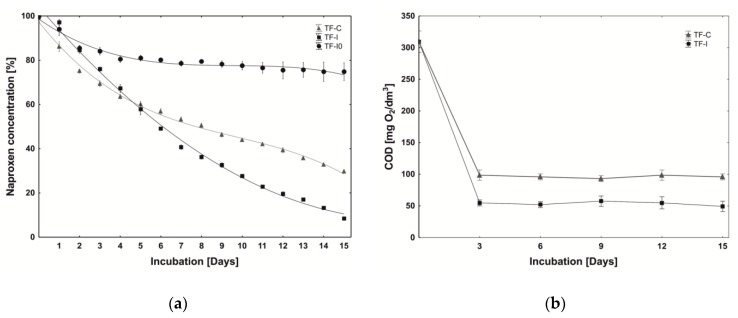
The trickling filters (TF) performance: (**a**) naproxen (1 mg/L) biodegradation: TF-C (Δ), TF-I (□) and TF-I0 (o); (**b**) removal of the COD in TF-C (Δ), TF-I (□).Data are presented as the mean ± the standard deviation of three replicates.

In the TF-I0 system, which only contained the lightweight expanded clay aggregate (LECA) and the microflora from the Imhoff tank, there was an almost 20% loss of the drug (Figure 1a) in the first four days, which was caused by its adsorption by the LECA and not because of biodegradation. Over the following days, naproxen concentration was constant. In the TF-C control system that contained both the LECA and immobilised B1(2015b) cells on the loofah sponges, 70% of the drug was removed. In the TF-I tricking filter with introduced B1(2015b) cells on the loofah sponges and the microflora from the Imhoff tank, almost 90% of the naproxen was degraded. These results indicate a synergistic interaction between introduced immobilized biocatalyst and autochthonous microflora on naproxen biodegradation.

At the same time that the naproxen biodegradation was being monitored in the trickling filters, the level of chemical oxygen demand (COD) was also determined. Samples for this analysis were taken from the collection tanks before the supplementation with the nutrients and glucose. After the stabilization processes, the decrease in the COD in TF-C and TF-I remained at a constant level (Figure 1b). More efficient decomposition of the organics from the synthetic wastewater was observed in TF-I (82.65 ± 1.01%) compared to TF-C (68.85 ± 0.074%). This was caused by the addition of microorganisms into TF-I that were specialized for wastewater treatment. The obtained results indicated that despite the relatively short time period (21 days), the stabilization processes were performed successfully and a fully functioning biofilm in the TF was developed.

### 2.3. Colonization of the Loofah Sponges

**Figure 2:**
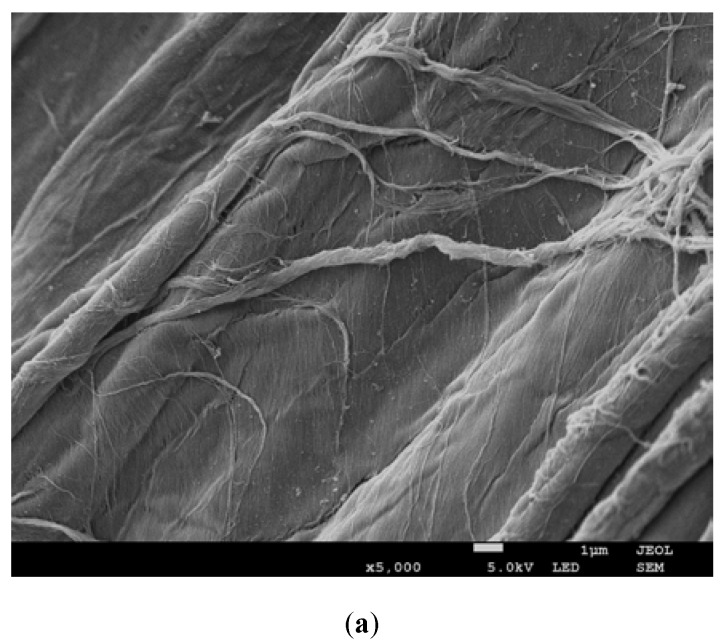

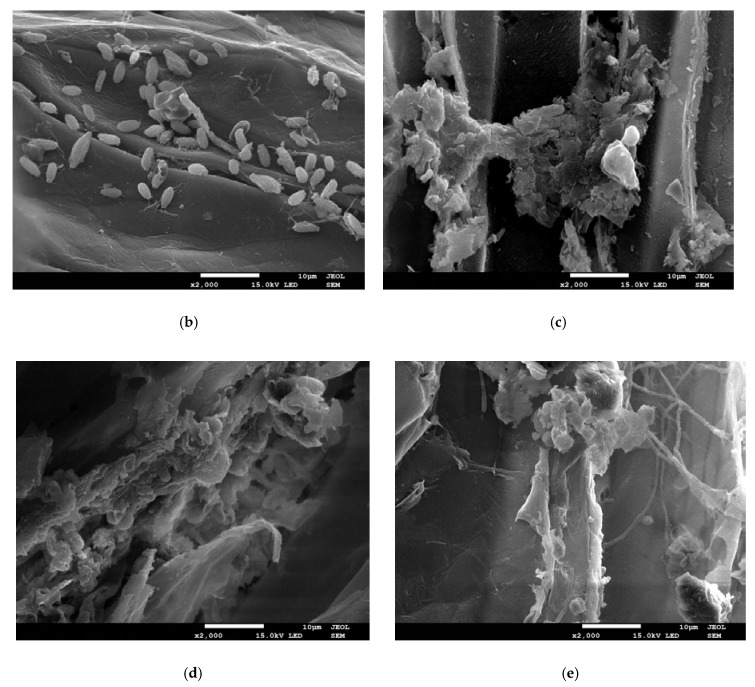
Scanning electron microscopy (SEM) micrographs of the uncolonized loofah sponges (**a**), that were colonized by *Bacillus thuringiensis* B1(2015b) (**b**), microflora from the Imhoff tank at the bottom (**c**), in the middle (**d**) and at the top (**e**) of the trickling filter.

Observation of the loofah sponges using scanning electron microscope (SEM) showed their highly porous structure, which was a suitable site for the attachment of the *Bacillus thuringiensis* B1(2015b) cells (Figure 2a). At the beginning of the experiment, immobilised B1(2015b) cells were clearly visible on the surface of the loofah sponges (Figure 2b). 

After 15 days, the loofah sponges that remained at the top, in the middle and at the bottom of the trickling filter were almost completely covered by the microflora from the Imhoff tank flow chamber (Figure 2c–e). The biofilm that had formed on the surface of the sponges was characterized by a large amount of extracellular matrix, and therefore detecting individual cells was very difficult. There were differences in the formation of the biofilm depending on the site in the trickling filter into which the immobilised strain was incorporated. We observed that bacterial biofilm was formed in the lower and the middle parts of the trickling filters, while fungal hyphae were dominant in the upper parts (Figure 2c–e).

### 2.4. Phylogenetic Characterization of the TF Microbial Population

**Figure 3:**
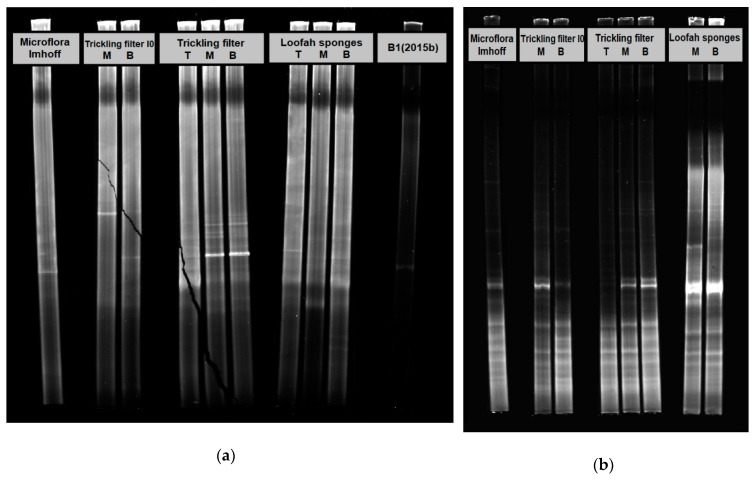
The denaturing gradient gel electrophoresis (DGGE) profiles that were obtained at different stages of the experiment using the amplified (**a**) bacterial V3-V5 regions of the 16S rRNA gene and (**b**) the fungal ITS1/2 regions of the 18S rRNA gene. The lines that are marked as Microflora Imhoff contain the sequences that were obtained from the middle part of TF-I0 before the exposure to naproxen; Trickling filter I0 after the exposure to naproxen; Trickling filter and loofah sponges after the complete biodegradation of naproxen; B1(2015b) contains the sequences that were obtained from pure cultures of B1(2015b) cells. The letters T M B represent the sampling sites: T—top, M—middle and B—bottom of the TF.

In this study, we analyzed the qualitative changes in both the bacterial (V3-V5 regions of the 16S rRNA gene) and fungal populations (ITS1/2 regions of the 18S rRNA gene) after acute, short-term exposure to naproxen (1 mg/L). As can be seen in Figure 3a, the untreated microflora from the Imhoff tank flow chamber was formulated by different bacterial strains with the *Pseudomonas* species being the dominant group. After 15 days of microflora exposure to naproxen, there were significant changes in the qualitative composition and a decrease in the diversity index (Table 2). The dominant groups of microorganisms that had been observed before the exposure to naproxen had probably the highest sensitivity to the drug, except for the strains that belong to the *Clostridium* sp. Interestingly, the presence of the drug caused a significant growth of the aerobic bacterium *Chryseolinea* sp. (Basic Local Alignment Search Tool (BLAST) similarity 100%), which to date has been found in soils in China and Korea and in Europe only in Germany. Although this genus is characteristic for uncontaminated forest soils, its tolerance to polycyclic aromatic hydrocarbons has not yet been studied. However, because this strain was no longer observed in the system after the bioaugmentation, it could be limited by other strains.

The fungal strains demonstrated a greater tolerance to naproxen than the bacteria. Changes in their composition, dominant groups and diversity index were not significant after their exposure to naproxen, except in the upper part of the TF, which contained a smaller amount of the 18S rDNA sequences (Figure 3b; Table 2).

Among the identified species, it was possible to distinguish fungi that belong to the genera *Trichosporon* sp. and *Vanrija* sp. The vast majority of the fungal DNA sequences (9 of 13) that were obtained were not similar to the sequences in the NCBI library.

By analyzing the 16S rDNA sequence from the B1(2015b) cells and comparing them with the sequenced genetic material from the TF and loofah sponges, it was confirmed that the bioaugmentation had been performed successfully. The immobilised B1(2015b) cells on the loofah sponges that remained in the trickling filter after 15 days were still present not only on the loofah sponges, but also in the core of the trickling filters (BLAST similarity 99%) (Figure 3a).

Therefore, after the biodegradation of naproxen, the influence of the immobilised B1(2015b) cells on the composition of the microflora from the Imhoff tank flow chamber in the trickling filter was analyzed. A comparison of the bacterial composition in the TF after exposure to naproxen (TF-I0) and after bioaugmentation shows a significant increase in biodiversity as a result of the introduction of the immobilised B1(2015b) cells (Figure 3a; Table 2). Changes were also observed in the dominant groups (*Clostridium* sp. and *Pseudomonas* sp. new domination) as well as in the growth of the bacteria that were present prior to the drug exposure and were sensitive to it (e.g., *Pseudomonas* sp.). Additionally, strains that were below the detection level before bioaugmentation were also present, probably as a result of the changes in the community composition. Analysis of the qualitative composition of the bacterial population that was introduced onto the loofah sponges indicated that the carrier was a good site for colonization. This was confirmed by the presence of strains from the LECA on the surface of the carrier. 

The lack of significant changes in the fungal community in this study indicates that bioaugmentation did not affect the composition of autochthonous strains or their diversity on the basic carrier (LECA) in the trickling filter (Figure 3b; Table 2). However, significant changes were observed in the number of fungal strains that colonized abundantly lignocellulosic sponges.

## 3. Discussion

### 3.1. Immobilization of Bacillus thuringiensis B1(2015b) on Loofah Sponge

The ability of bacteria to form a biofilm on the surface of various materials can provide many advantages for cells such as a higher level of resistance to the toxic compounds in the environment. In bioremediation, a biofilm matrix additionally ensures a better chance for the adaptation and survival of bacteria in a new environment with autochthonic microflora. Because the efficiency of bioremediation using a trickling filter depends on the quality of the biofilm that is formed, its parameters for immobilization were optimized by adsorption on loofah sponge. Among the various accessible materials that are used for immobilization, it is desirable that carriers should be biodegradable and biocompatible when being used for bioremediation. At the same time, carriers have to also be characterized by a high level of porosity, mechanical resistance and a low price. Loofah sponges, which are composed of cellulose, hemicellulose and lignin, are an eco-friendly material for bacterial cell immobilizations due to their high mechanical resistance and high porosity [8,9].

The *Bacillus thuringiensis* B1(2015b) that was used in this study was isolated from the soil of the chemical factory “Organika-Azot” in Jaworzno, Poland. This Gram-positive strain is able to degrade various aromatic compounds such as phenol, vanillic acid, protocatechuic acid, benzoic acid or 4-hydroxybenzoic acid as well as some non-steroidal anti-inflammatory drugs such as naproxen and ibuprofen [6,10]. Because of these valuable features, this strain was immobilised on the loofah sponge.

One of the major factors that influences attachment is the surface charge of bacterial cells, which depends on the positively or negatively charged molecules that are predominant on the surface of the bacterial cell wall. Most bacteria have a negatively charged surface (negative zeta potential) at physiological pH (pH 7). However, the bacteria cell wall zeta potential is strongly determined by the ionic strength of the surrounding medium and pH value [11]. It has been shown that *Bacillus cereus* spores exhibit a higher efficiency of attachment to hydrophobized, negatively charged glass when the medium has the same pH as the isoelectric point of their cell wall (pH 3) [12]. Due to the fact that loofah sponge is composed of polysaccharides, its charge at pH equal to or higher than 7 is negative. For that reason, the same charge of the cell surface and the carrier complicates the initial cell adhesion to the carrier surface. High ionic strength facilitates the neutralization of the cell wall charge when the cell and carrier surfaces have the same charge and in this way, it reduces their electrostatic repulsion [11].

It is known that adding manganese significantly increases the amount of the biofilm of *Bacillus* species [13]. Manganese belongs to the group of essential metals that regulate the activity of many enzymes by functioning as a catalytic cofactor. One of these enzymes is phosphoglycerate phosphomutase (EC 5.4.2.1), which plays a crucial role during the initiation of sporulation. This enzyme is responsible for the accumulation of 3-phosphoglyceric acid (3-PGA) in spores and its utilisation during germination [14]. On the other hand, other studies have indicated that manganese is complexed with Spo0F (a signal mediator), which is involved in the sporulation phosphorelay. In this mechanism, the phosphate is transferred from histidine kinase to Spo0F and later to Spo0B (the phosphotransferase). The final step is the transition of the phosphate to Spo0A, which is a transcription factor that initiates spore or biofilm formation [13,15]. Simultaneously, an increase in the amount of phosphorylated Spo0A results in unblocking the sinI transcription. SinI subsequently represses the activity of SinR and thereby prevents the blocking of the *eps* and *tapA* operons, which are involved in exopolysaccharide (EPS) and TasA protein (one of the matrix components) synthesis, respectively [16]. Despite the fact that a HCT medium already contained manganese (0.015 mM), during the optimization of the immobilization conditions, the addition of 0.33 mM of manganese significantly improved the quality of biofilm that was formed by the B1(2015b) cells. It is noteworthy that Morikawa et al. [17] showed that even at a concentration of 1000 mM, manganese stimulated biofilm formation by the *Bacillus subtilis* B1 strain.

### 3.2. Naproxen Biodegradation in the Trickling Filters

Because of its polycyclic structure, naproxen belongs to the group of hard-to-biodegrade xenobiotics. However, naproxen removal has been observed in monoculture conditions by only a few bacterial strains [4,6]. Moreover, Górny et al. [10] proposed a naproxen biodegradation pathway by *Bacillus thuringiensis* B1(2015b). According to their studies, the first stage comprises demethylation by tetrahydrofolate-dependent *O*-demethylase, which was observed so far only during fungal biotransformation. In the next step, *O*-desmethylnaproxen probably undergoes hydroxylation to 7-hydroxy-*O*-desmethylnaproxen. Due to the fact that naproxen is naphthalene derivative, it might be degraded like naphthalene however, only 2-formyl-5-hydroxyphenylacetic acid was detected. The last observed intermediate was salicylic acid which is transformed into maleyl puryvate, 2-oxo-3,5-heptadienedioic acid or *cis,cis-*muconic acid. These results indicated that strain *Bacillus thuringiensis* B1(2015b) is able to degrade naproxen into the tricarboxylic acid cycle intermediates. The ability of strain B1(2015b) to naproxen mineralization has made it a promising strain for the complete removal of this drug from wastewater.

On the other hand, only a few studies have been done with mixed microflora under non-sterile conditions. Due to the overgrowth of autochthonous bacteria and the inhibition of the growth of the microorganisms that are able to degrade these types of contaminants, the removal efficiency of naproxen was significantly lower than in monoculture conditions [18]. Because of the growing problem of drugs in the environment, there is a need to develop bioremediation methods adapted to their removal.

One of the key elements of a trickling filter is the carrier on which the biofilm is created. To date, various packing materials, such as plastics, stones [19], polyurethane foam [20] or zeolite have been used [21]. However, by selecting the correct packing material, it is possible to improve the effectiveness of the performance of a trickling filter. In this study, we selected lightweight expanded clay aggregate (LECA) (particle size 10–20 mm) due to its high porosity, natural origin, low cost and lack of toxicity. Additionally, studies on the usability of LECA in bioremediation have revealed its high sorption properties for heavy metals and certain pollutants [22].

In order to create a fully functioning trickling filter, there was a need to implement its by biological active microflora. The TF-I and TF-I0 systems that were constructed for this study were inoculated with microflora from the Imhoff tank flow chamber from the wastewater treatment plant of Krupski Młyn-Ziętek (Poland), which was built almost 60 years ago and has never been modernized or otherwise augmented since that time.

The TF-I0 system was constructed in order to examine if the autochthonous microflora from the Imhoff tank flow chamber was able to biodegrade naproxen. The conducted study revealed that the initial drug loss was caused by LECA adsorption and not as an effect of biodegradation. It was confirmed in an additional control system, which contained only LECA and synthetic wastewater contaminated with naproxen (results not shown). This result shows that even wastewater treatment system with almost 60 years of history do not have microflora able to degrade or remove naproxen. Fungi primarily have the ability to degrade non-steroidal inflammatory drugs, which also implies that they are less sensitive to these compounds. This is related to the presence of more advanced enzyme complexes and detoxification pathways than those that are observed in bacterial strains [23]. We could suppose that the presence of fungi in bioremediation systems will provide a higher possibility of xenobiotic degradation for drugs such as NSAIDs. However, fungi only constituted a small percentage of the entire microflora of the trickling filters and activated sludge.

After introducing the immobilized cells of *Bacillus thuringiensis* B1(2015b) into the trickling filters, they were able to degrade naproxen in the presence of the autochthonous microflora and beyond their optimal growth conditions. Moreover, we observed a positive influence of the synergism between the introduced B1(2015b) strain and the autochthonous microflora on naproxen biodegradation. Although this type of ecological interactions occurs often in bioremediation systems, their mechanisms of action are not well known. It is assumed that this might be caused by increasing the bioavailability of a contaminant through the production of surfactants, a metabolic association of intermediates that cannot be further degraded, an exchange of growth factors or enhanced aggregation. For example, Byss et al. [24] observed that the inoculation with *Pleurotus ostreatus* of soil that had been contaminated with PAH resulted in significant stimulation of the growth of G+ bacteria and simultaneously more efficient bioremediation. In our study, we suspected that synergisms could be caused by the exchange of growth factors and the interaction of the Imhoff population and the B1(2015b) strain. Additionally, the presence of the anaerobic bacterial communities in the lower part of the TF (e.g., *Clostridium* sp.), could accelerate the naproxen biodegradation, due to the ability to demethylation or degradation of aromatic compounds like veratrate or catechol [25]. However, the B1(2015b) cells were able to degrade naproxen.

A further major factor that affects the performance of attached-growth bioreactors is the selection of an appropriate carbon to nitrogen (C:N) ratio in synthetic wastewater, which is associated with ensuring adequate amounts of carbon compounds, which are electron donors in the denitrification process. Under carbon-deficit conditions (C:N 3:1), nitrification and denitrification rates may not be in equilibrium and as a result, there is a lower performance of the bioreactors [2]. In our study, we prepared synthetic wastewater with a C:N ratio of 10:1, which according to Xia et al. [2], provides the best efficiency for the reduction of the COD in biofilm-based bioreactors. Additionally, immobilized cells of B1(2015b) introduced into the TF without autochthonous microflora were able to degrade most of the organic carbon from the synthetic wastewater. However, the addition of the specialized microflora from the wastewater treatment plant resulted in the higher removal of the organic carbon.

### 3.3. Colonization of the Loofah Sponges

In response to deficiencies of organic and mineral compounds, temperature and pH fluctuations, the presence of heavy metals as well as the substances that are secreted by other organisms, microorganisms began to aggregate into extensive communities in the form of biofilm. This solution ensured the most critical advantages in the context of the colonization of aquatic environments. Biofilm forms various structures and water channels, which provide mechanical stability, mass transport, and functional heterogeneity. However, the key element is the biofilm matrix, whose main component is a highly hydrated EPS. Because of its sorption properties, biofilm accumulates nutrients as well as the exopolysaccharides, lipids, nucleic acids and enzymes that are secreted by cells while it also limits the direct contact of cells with toxins [4,8,26]. In natural habitats, biofilm is composed of multiple, highly selected strains of microorganisms. This microbiological diversification ensures metabolic cooperation and genetic exchanges among the populations in the biofilm [26]. For example, a synergistic effect occurred during the testing of the sodium dodecyl sulfate (SDS) resistance of *Pseudomonas aeruginosa*, *P. protegens* and *Klebsiella pneumoniae* strains. In monospecies biofilms, strains had different levels of resistance, whereas in a consortium, the more resistant species protected the rest of the community [27]. In bioremediation systems consortium we can also observe a more abundant biofilm matrix, in which the microorganisms are exposed to higher concentrations of potentially toxic compounds and required a protective barrier [3].

A characteristic feature of trickling filters is the diversity of conditions depending on the height. This also results in a different variety of microorganisms at different levels which is connected with the occurrence of two regions—the upper part of the aerobic region and a progressive lower anaerobic region due to the lack of additional aeration [21].

### 3.4. Phylogenetic Characterization of the TF Microbial Population

In bioremediation systems, autochthonous microflora operates in the form of cooperating communities. However, the presence of factors such as pollution or the addition or depletion of nutrients can significantly affect the diversity of microbial communities. The impact of pollutants on diversity depends on its type and the duration of the exposure. It was demonstrated that naproxen can negatively affect non-target organisms and entire ecosystems [28]. To date, there have only been a few studies on the effects of naproxen on microbial communities. Grenni et al. [28] observed that after 3 h of naproxen exposure (100 μg/L), the number of live cells of the microorganisms from the Tiber River decreased drastically. At a concentration of 10 μM, naproxen also inhibits nitrite production by the ammonia-oxidizing bacterium (AOB) *Nitrosomonas europaea,* which is a fundamental member of the microflora in wastewater treatment systems [29].

However, little is known about how naproxen affects the compositions of the microorganisms communities in bioremediation systems that are not adapted to degrade NSAIDs. In this study, we observed significant changes in the bacterial community which resulted in a drastic reduction in their biodiversity. This result shows how much risk naproxen can pose to bacteria in wastewater treatment plant if it enters in large quantities.

It should be also stressed that bioaugmentation is not indifferent to the autochthonous microflora in a bioremediation system. This effect can be positive or negative. The presence of new strains can change the composition of the microbial community through competition or inhibition. In the case of immobilization, the carrier onto which a strain is introduced should also be considered to be an influencing factor.

A carrier that can be a carbon and energy source for a specific group of microorganisms should be introduced carefully. A too large amount can cause these species to be too dominant, which could disrupt the performance of the entire system. In this study, we analyzed the influence of bioaugmentation over a short period of time and after 15 days, the beginning of overgrowth was observed. Further analyses should be performed to assess the long-term impact of the introduction of cellulosic materials into the communities in wastewater treatment systems.

## 4. Materials and Methods

### 4.1. Immobilization Optimisation and Procedure

In order to develop an immobilisation procedure that will results in the most abundant biofilm, ten immobilization parameters for optimization were accessed. These included the type of growth medium used: nutrient broth, mineral salts medium, HCT medium and its supplementation with glucose (0.5g/L). Additionally, we also assess the age of the culture that was harvested for immobilization (24, 48 or 72 h) and initial culture optical density (0.2, 0.4, 0.6, 0.8, or 1.0). The influence of the agitation speed (70, 90, 110, 130 or 150 rpm), incubation time of the bacterial culture with carrier (16, 24, 48 or 72 h), temperature (15, 20, 25, 30, or 35 °C), medium pH (3, 4, 5, 6, 7.2 or 8), salt concentration (7, 8, 14, 17, 24 or 34 g/L) and additional supplementation with metal salts (manganese, iron or calcium) was studied.

The final procedure used after immobilization was as follows: the bacterial strain *Bacillus thuringiensis* B1(2015b), which was cultivated according to Marchlewicz et al. [6] and re-suspended in the HCT medium, was used for the immobilization [7]. The optical density value (OD_600_) of the bacterial suspension that was prepared for the immobilization was equal to 0.2. Loofah sponges, which were prepared according to Dzionek et al. [4], were used as the carrier for the immobilization of the B1(2015b) cells. For the immobilization, each Erlenmeyer flask (1000 mL) that contained sterile carrier material (7.5 g) was inoculated with the bacterial cell suspension (600 mL). The HCT medium (pH 8) in which the immobilization process was conducted was additionally supplemented with glucose (0.5 g/L) and manganese sulphate (1 g/L). The flasks were incubated with shaking (110 rpm) at 20 °C for 48 h. After incubation, loofah sponges with the immobilised B1(2015b) cells were rinsed with NaCl (0.9%) in order to remove any unbound microorganisms and were then used for bioremediation experiments.

### 4.2. Configuration and Operational Conditions of the Trickling Filters (TFs)

**Figure 4:**
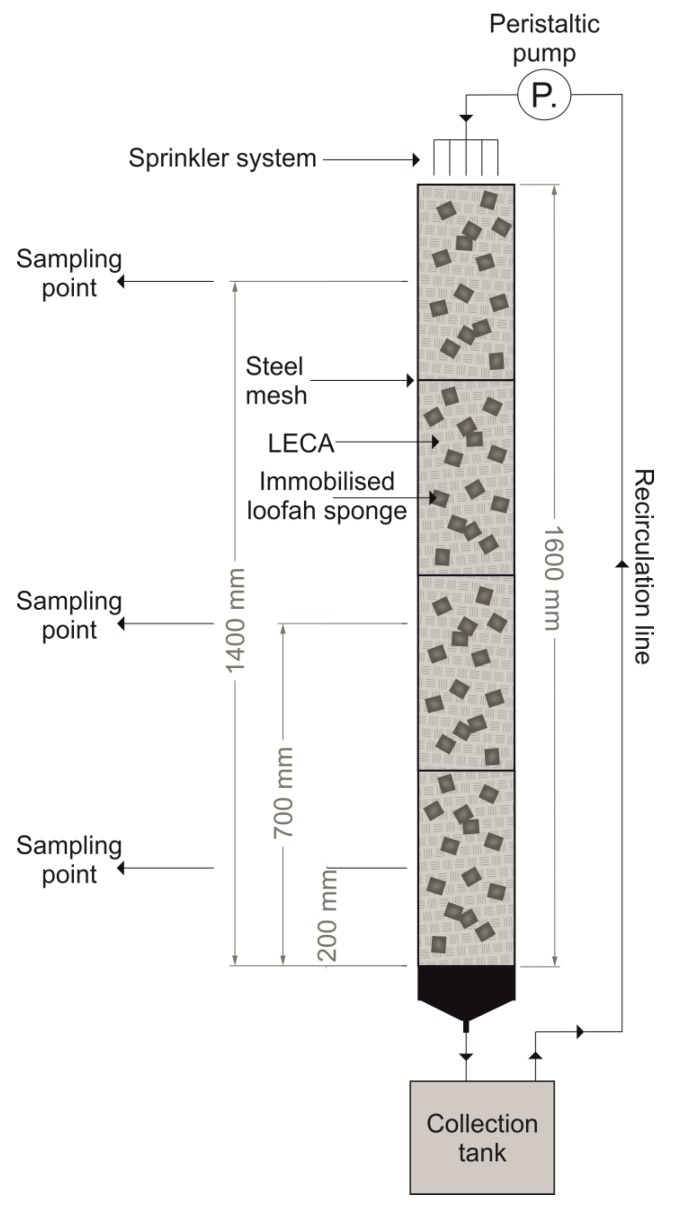
Experimental set-up of the trickling filters.

Three lab-scale TFs were constructed for this study, namely TF-I, TF-C, and TF-I0. Each TF was composed of four filter units. One filter unit consisted of polyvinyl chloride (PVC) pipe (H × W = (400 × 100 mm) × 4), which was protected from the bottom by steel mesh and filled with the biofilm carriers. The total volume of each TF was 0.015 m^3^ and the filling was 46% of the reactor volume. As a base, biofilm carriers were selected lightweight expanded clay aggregate (LECA), which constituted 70% of each filter unit filling volume. The remaining 30% of the filling comprised the loofah sponges with immobilized bacteria (Figure 4).

As the nutrient and carbon sources, synthetic wastewater (15 L) was continuously circulated into the trickling filters by a peristaltic pump with a flow rate of approximately 0.0066 m^3^/h. The synthetic wastewater was based on the one that was proposed by Kosjek et al. [30] with some modifications. The composition per 1 L was as follows: 0.317 g CH_3_COONH_4_; 0.04 g NH_4_Cl; 0.024 g K_2_HPO_4_; 0.008 g KH_2_PO_4_; 0.1 g CaCO_3_; 0.2 g MgSO_4_ × 7H_2_O; 0.04 g NaCl; 0.005 g FeSO_4_∙× 7H_2_O and 0.6 g glucose. A nutrient and glucose stock solution were added to the collection tanks every three days. The addition of calcium carbonate prevented the excessive acidification of the wastewater, whose pH was adjusted to 7.6 and was inspected every three days. The temperature in the trickling filters was maintained within a range of 21–23 °C. The hydraulic residence time (HRT) was determined using a draining test and is expressed as the ratio of the volume of the liquid in the trickling filter to the volumetric flow rate [31]. The chemical oxygen demand (COD) was analyzed using the potassium dichromate method according to the standard procedures [32].

Trickling filter TF-I was inoculated with biomass that had been taken from the Imhoff tank flow chamber in the wastewater treatment plant of Krupski Młyn-Ziętek (Poland). To colonize the trickling filter, a mixture of biomass and synthetic wastewater was continuously circulated through TF-I. When the biofilm on the surface of the LECA reached a thickness of 2–3 mm, the stabilisation of TF-I was considered to be complete (21 days) and loofah sponges with the immobilized bacteria were added to each filtration unit. The first control trickling filter (TF-C) contained only loofah sponges with immobilized bacteria and the LECA. The second control trickling filter (TF-IO) contained only stabilised biomass (2–3 mm thick biofilm) from the Imhoff tank flow chamber and LECA.

### 4.3. Naproxen Biodegradation Experiments

Fresh synthetic wastewater (15 L) that had been supplemented with naproxen (1 mg/L) was added to each trickling filter and circulated for the next 15 days. All of the experiments were conducted in a closed circuit. To determine the naproxen concentration, synthetic wastewater samples were taken from the collection tanks every 24 h for 15 days and analyzed using HPLC equipped with a LiChromospher^®^ RP-18 column (4 × 250 mm), liChroCART^®^ 250-4 Nucleosil 5 C18 and a DAD detector (Merck HITACHI, Darmstadt, Germany). As a mobile phase was used acetonitrile and 1% acetic acid (50:50 *v/v*) at a flow rate of 1 mL × min^−1^. Naproxen was detected in the supernatant at wavelength 260 nm. Identification and quantification by comparison the HPLC retention times and UV-visible spectra with the external standards were conducted [23].

Uninoculated, additional controls (contained only LECA or synthetic wastewater supplemented with naproxen) were also prepared in order to determine adsorption or abiotic degradation of the drug.

To investigate the microbial population on the LECA and loofah sponges, the carriers were taken from three sampling points at depths of 20, 700 and 1400 mm (Figure S1, Supplementary Materials). To examine the effect of naproxen on the microflora from the Imhoff tank flow chamber (TF-I0), the LECA samples were taken before the naproxen was added and after 15 days of runoff. In order to investigate the colonization of the loofah sponges by the microflora from the Imhoff tank flow chamber, samples of the loofah sponges were taken after the complete biodegradation of naproxen (TF-I).

### 4.4. Biofilm Analysis Using Scanning Electron Microscopy

The biofilm structure on the loofah sponges was observed before and after naproxen biodegradation in trickling filter TF-I using Scanning Electron Microscopy. Samples were prepared according to Dzionek et al. [4] and were observed with a JSM-7100F TTL LV high-resolution electron microscope (JEOL, Tokyo, Japan).

### 4.5. Phylogenetic Characterization of the Microbial Population in the TFs

Changes in the microbial population on the carriers were determined using the denaturing gradient gel electrophoresis (DGGE) method. In order to obtain the DNA samples, the biofilm from the carrier samples was removed by shaking and rinsing with a NaCl solution (0.9%), centrifuged (14,000 rpm, 20 min) and re-suspended in the same solution. DNA was immediately extracted from various materials using a Genomic DNA isolation kit (A&A Biotechnology, Gdynia, Poland) according to the manufacturer’s instructions.

The PCR amplification of the bacterial V3-V5 region of 16S rRNA gene (about 570 bp) was performed using the universal primers MF341-GC (5′-CGC CCG CCG CGC CCC GCG CCC GTC CCG CCG CCC CCG CCCG CCT ACG GGA GGC AGC AG-3′) and MR907 (5′–CCG TCA ATT CMT TTG AGT TT-3′) [33]. In order to obtain the sequences of the highly conserved regions of ITS1 and ITS2 from the fungal rRNA gene (500–800 bp), the primers ITS1F (5′–CTT GGT CAT TTA GAG GAA GTAA-3′) and ITS4 (5′–TCC TCC GCT TAT TGA TAT GC-3′) were used [34]. Each 25 μL PCR reaction contained 1 μL of extracted DNA, a 1 × PCR buffer, a 10 mM dNTP mixture, 10 mM of forward and reverse primers, 0.2 mg/L BSA and 1.25 U Pfu DNA Polymerase. Amplification was carried out with the cycling conditions for bacterial 16S rDNA according to Płociniczak et al. [33] and Anderson et al. [34] for the fungal ITS. The PCR products were examined with a 1.5% agarose gel in order to isolate the DNA fragments of the required length and to reamplify them.

DGGE was performed with the D-code System (Bio-Rad, Hercules, CA, USA) according to Płociniczak et al. [33]. The PCR products were loaded directly onto 6% (for 16S rRNA gene) or 8% (for ITS regions) polyacrylamide gels in a 1 × TAE buffer. The gels were prepared with a denaturing gradient in the range of 30–60% (for the 16S rRNA gene) or 18–58% (for the ITS regions). The electrophoresis was first run at 180 V for 30 min and then at 80 V for 17 h at 60 °C. The gels were subsequently stained with SYBR Gold (Thermo Fisher Scientific, Waltham, MA, USA) according to the manufacturer’s instructions. The strong bands were cut out and diluted in 25 μL sterile water overnight, reamplified with the primers described above (except for primer MF341-GC, which was used without a GC clamp) and sequenced. The nucleotide sequences that were obtained were compared with known sequences in GenBank using the BLAST program (https://blast.ncbi.nlm.nih.gov).

The intensity of the individual DGGE bands was evaluated using ImageJ software and was scored as absent (value 0) or present on a scale of 1 to 4 in order to generate a data set. The diversity of a microbial population was determined using the Shannon Wiener index (*H*’) according to Xia et al. [2].

### 4.6. Statistical Analysis

All of the experiments were performed in at least three replicates. The values of the efficiency of naproxen biodegradation and the values of the physico-chemical parameters that were obtained were analyzed using the STATISTICA 12 PL software package (StatSoft, Tulsa, OK, USA). Statistically significant differences and similarities were determined using the t-test or the Least Significant Differences (LSD) test (*p* ≥ 0.05).

## 5. Conclusions

The immobilised *Bacillus thuringiensis* B1(2015b) introduced onto loofah sponges were successfully incorporated into the trickling filters. Synergistic influence of the autochthonous microflora on the naproxen biodegradation that was performed by the immobilised B1(2015b) cells was revealed. This short-term analysis revealed the possible effects of introducing a larger quantity of naproxen into a wastewater treatment plant. One of them can be a large decrease in the microbial diversity.

## Figures and Tables

**Table 1:** Comparison of the *B. thuringiensis* B1(2015b) biofilm mass that was created on loofah sponge and its metabolic activity before and after the optimization of the immobilization process.

Development Stage	Dry Biofilm Mass [mg]	Fluorescein Concentration [μg·mL^−1^]	Total Enzymatic Activity [μg·g Dry Mass^−1^·h^−1^]
Before optimization	8.3 ± 0.9	4.56 ± 0.48	532.77 ± 39.09
After optimization	28 ± 3.5	19.07 ± 1.06	709.14 ± 40.60

**Table 2:** Changes in the Shannon-Wiener index (*H’*) of the bacterial (16S rDNA) and fungal (18S rDNA) communities corresponding to the denaturing gradient gel electrophoresis (DGGE) profiles that were obtained after the treatments. Columns marked as Microflora Imhoff contain the *H*’ values that were calculated from the middle part of TF-I0 before the exposure to naproxen; Trickling filter I0 after the exposure to naproxen; Trickling filter and the loofah sponges after the complete biodegradation of naproxen. The letters T M B represent the sampling sites: T—top, M—middle and B—bottom of the TF.

	Microflora Imhoff	Trickling Filter I0		Trickling Filter			Loofah Sponges		
		M	B	T	M	B	T	M	B
**16S rDNA**	2.164	1.039	1.386	2.025	2.307	2.253	1.886	1.791	2.342
**18S rDNA**	2.686	2.761	2.043	2.564	2.780	2.718	-	2.800	2.841

## Supplementary Materials

The following are available online: Figure S1: Influence of different variants of environmental and physiological factors on the immobilisation efficiency of *Bacillus thuringiensis* B1(2015b) cells on loofah sponges.
