# Supplementary material for: Enhanced Degradation of Naproxen by Immobilization of Bacillus thuringiensis B1(2015b) on Loofah Sponge
Source: Molecules. 2020 Feb 17;25(4):872. doi: 10.3390/molecules25040872 (PMC7070439; doi:10.3390/molecules25040872)
Supplement: Supplementary file 1 [file molecules-25-00872-s001.pdf]

Supplementary Material

Enhanced degradation of naproxen by immobilization of *Bacillus thuringiensis* B1(2015b) on loofah sponge

Anna Dzionek[1], Danuta Wojcieszyńska[1], Małgorzata Adamczyk-Habrajska[2] and Urszula Guzik[1*]

[1] University of Silesia in Katowice, Faculty of Natural Science, Institute of Biology, Biotechnology and Environmental Protection, Jagiellońska 28, 40-032 Katowice, Poland
[2] University of Silesia in Katowice, Faculty of Science and Technology, Institute of Materials Engineering, 75 Pułku Piechoty 1a, 41–500 Chorzów, Poland

* Correspondence to: urszula.guzik@us.edu.pl; Tel.: +32-2009-567

Contents:

Figure S1: Influence of different variants of environmental and physiological factors on the immobilisation efficiency of *Bacillus thuringiensis* B1(2015b) cells on loofah sponges.

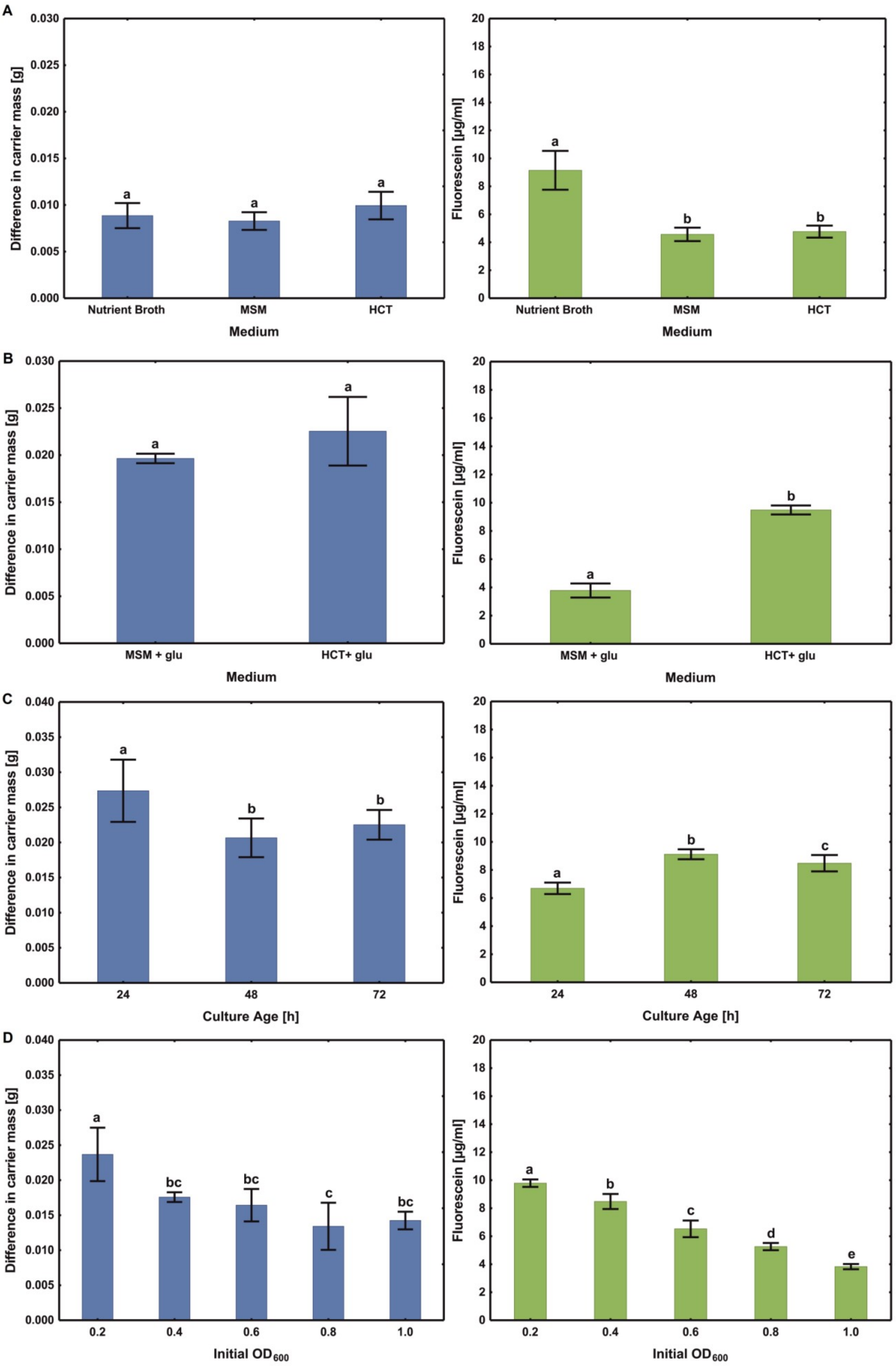
A
B
C
D
Difference in carrier mass [g]
Fluorescein [µg/ml]
Nutrient Broth
MSM
HCT
Medium
MSM + glu
HCT+ glu
Culture Age [h]
24
48
72
Initial OD600
0.2
0.4
0.6
0.8
1.0
a
b
c
d
e
bc

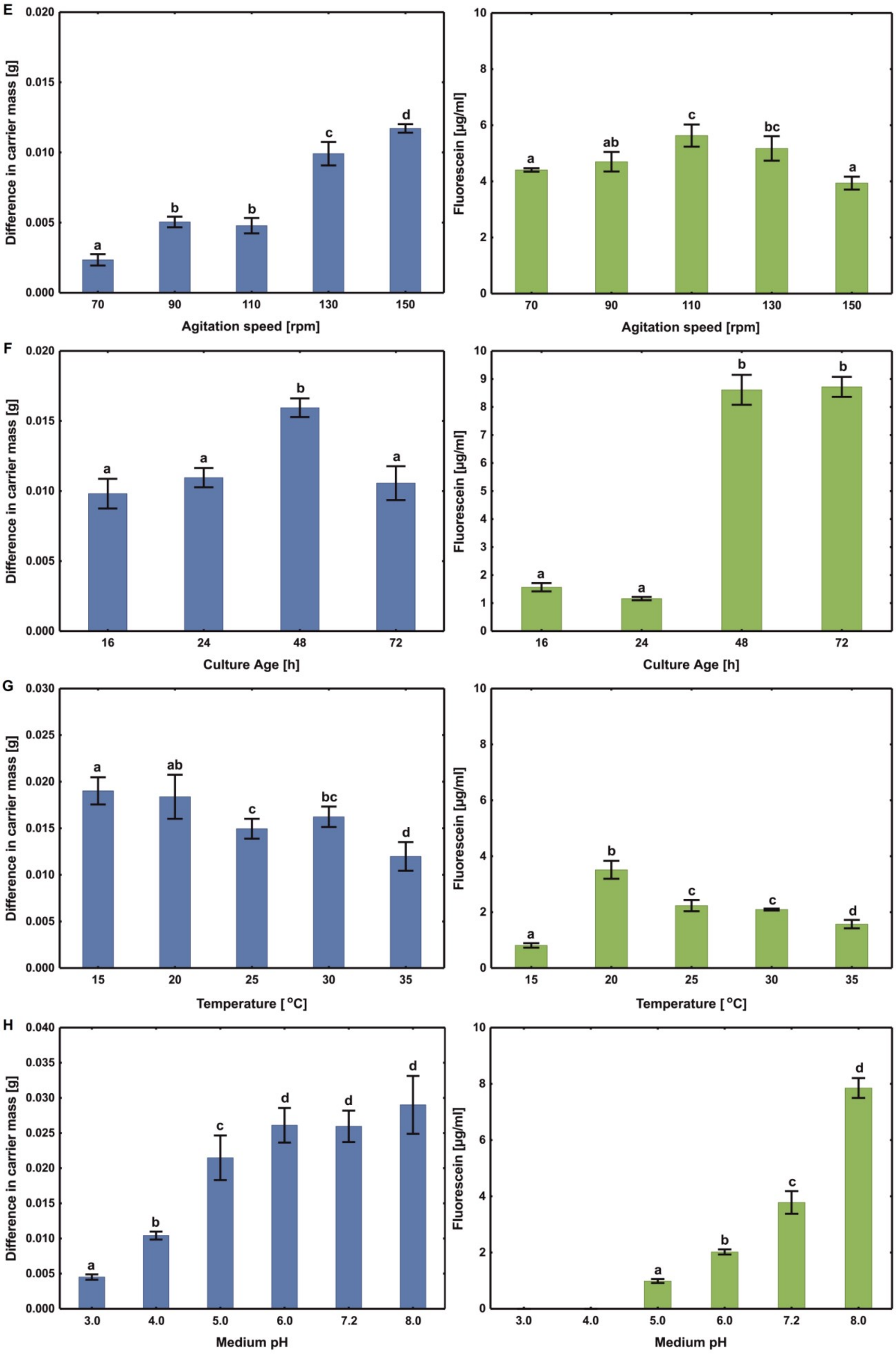
E
Difference in carrier mass [g]
Agitation speed [rpm]
70
90
110
130
150
a
b
b
c
d
Fluorescein [µg/ml]
Agitation speed [rpm]
a
ab
c
bc
a
F
Difference in carrier mass [g]
Culture Age [h]
16
24
48
72
a
a
b
a
Fluorescein [µg/ml]
Culture Age [h]
a
a
b
b
G
Difference in carrier mass [g]
Temperature [°C]
15
20
25
30
35
a
ab
c
bc
d
Fluorescein [µg/ml]
Temperature [°C]
a
b
c
c
d
H
Difference in carrier mass [g]
Medium pH
3.0
4.0
5.0
6.0
7.2
8.0
a
b
c
d
d
d
Fluorescein [µg/ml]
Medium pH
a
b
c
d

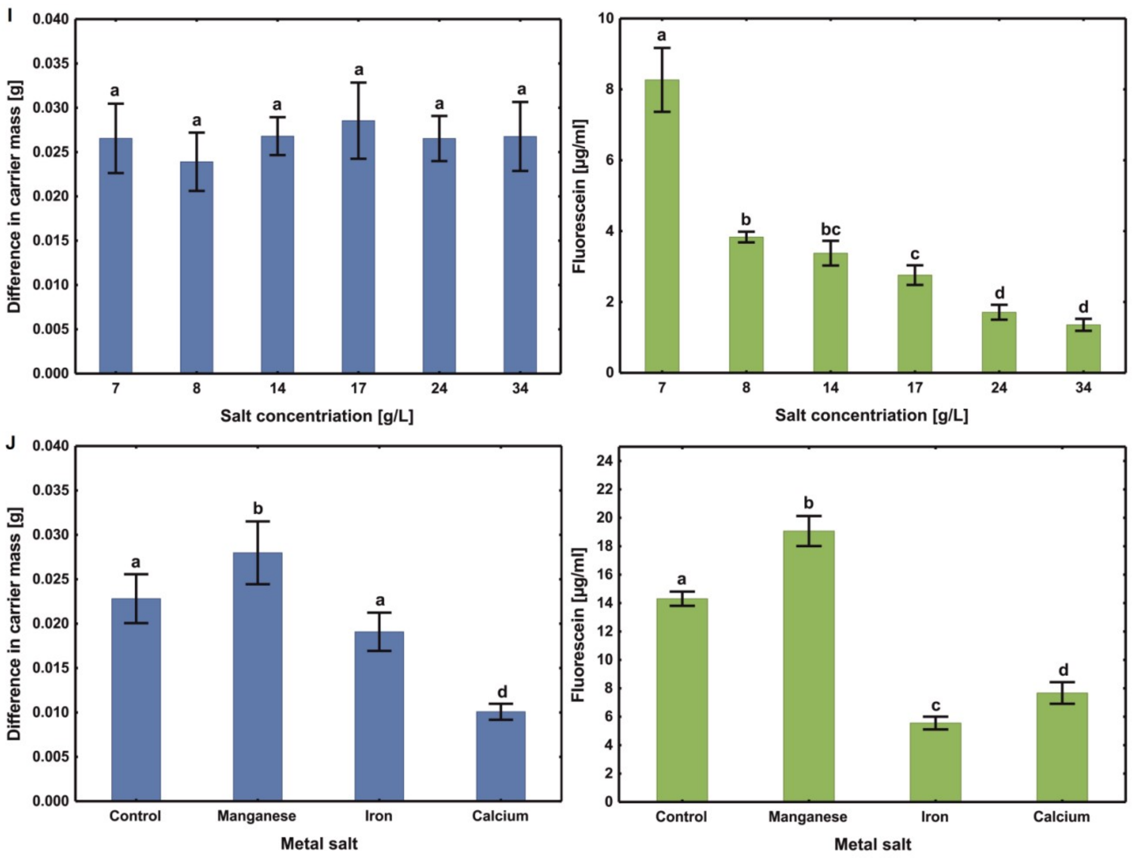

Figure S1: Influence of environmental and physiological factors on the immobilisation efficiency of *Bacillus thuringingiensis* B1(2015b) cells on loofah sponges:

(**a**) Immobilisation medium (Nutrient Broth, Mineral Salts Medium or HCT (medium rich in both carbon and ion sources));
(**b**) Additional carbon source (Mineral Salts Medium + 0.5 g/L glucose or HTC + 0.5 g/L glucose);
(**c**) Age of the culture that was harvested for immobilisation (24, 48 or 72 h);
(**d**) Initial culture Optical Density (0.2, 0.4, 0.6, 0.8, or 1.0);
(**e**) Agitation speed (70, 90, 110, 130 or 150 rpm);
(**f**) Incubation time of the bacterial culture with a carrier (16, 24, 48 or 72 h);
(**g**) Temperature (15, 20, 25, 30, or 35°C);
(**h**) Immobilisation medium pH (3, 4, 5, 6, 7.2 or 8);
(**i**) Salt concentration in the immobilisation medium (7, 8, 14, 17, 24 or 34 g/L);
(**j**) Additional metal ions (manganese, iron or calcium).

All experiments were performed in at least five replicates. The values of the efficiency of immobilisation and enzyme activities were analyzed using a one-way ANOVA ($p \geq 0.05$) using STATISTICA 10 PL software package. A post-hoc test or T-test was applied to assess the differences between the treatments (differences are marked with successive letters a, b, c, d or e).
